# Polish Cross-Cultural Adaptation of the Lower Limb Functional Index (LLFI) Demonstrates a Valid Outcome Measure for the Lower Limb Region and Joints

**DOI:** 10.3390/ijerph18189894

**Published:** 2021-09-20

**Authors:** Agnieszka Bejer, Agnieszka Bieś, Sylwia Kyc, Magdalena Lorenc, Piotr Mataczyński, Elżbieta Domka-Jopek, Markus Melloh, Charles Philip Gabel

**Affiliations:** 1 Institute of Health Sciences, Medical College of Rzeszow University, 35-959 Rzeszów, Poland; agnieszkapodufaly@gmail.com (A.B.); sylwia.kyc@gmail.com (S.K.); michalekmagdalena@wp.pl (M.L.); mataczynskipiotr@gmail.com (P.M.); edomka@ur.edu.pl (E.D.-J.); 2 The Holy Family Specialist Hospital, Rudna Mała, 36-060 Głogów Małopolski, Poland; 3 Faculty of Health, Victoria University of Wellington, Wellington 6140, New Zealand; markus.melloh@vuw.ac.nz; 4 School of Health Professions, Zurich University of Applied Sciences, 8401 Winterthur, Switzerland; 5 Curtin Medical School, Curtin University, Bentley, WA 6102, Australia; 6 UWA Medical School, University of Western Australia, Nedlands, WA 6009, Australia; 7 Access Physiotherapy, Coolum Beach, QLD 4573, Australia; cp.gabel@bigpond.com

**Keywords:** lower limb functional index, outcome measure, linguistic adaptation, psychometrics

## Abstract

This study aimed to perform linguistic and cross-cultural adaptation to establish a Polish version of the Lower Limb Functional Index (LLFI) as well as an evaluation of the psychometric properties. This was a two-stage, cross-sectional study. The first stage—linguistic and cultural adaptation, complied with the International Society for Pharmacoeconomics and Outcomes Research guidelines to produce the Lower Limb Functional Index, Polish version (LLFI-PL). The subjects were recruited to the second stage of the study from a sample of convenience (*n* = 125, age $\overset{-}{x}$ = 52.86 ± 19.53 years, 56% female, symptoms duration $\overset{-}{x}$ = 17.69 ± 18.39 weeks). Baseline reliability was performed on the LLFI-PL with retest period at 3–7 days. The Western Ontario and McMaster University Osteoarthritis Index (WOMAC), EuroQol Health Questionnaire 5-Dimensions 5-Level (EQ-5D-5L), and an 11-point Pain Numerical Rating Scale (P-NRS) were completed to assess the validity of the LLFI-PL. Statistical analysis showed high internal consistency (α = 0.94), and excellent test–retest reliability (ICC_2.1_ = 0.96). The measurement error was SEM = 1.69% with MDC_90_ = 3.93%. Construct validity demonstrated strong correlations between the LLFI-PL and WOMAC (r = 0.81) and moderate correlations with the EQ-5D-5L (*r* = −0.63) and P-NRS (*r* = −0.39). Exploratory factor analysis confirmed a single-factor structure. The LLFI-PL is a psychometrically sound questionnaire for Polish-speaking patients with lower limb musculoskeletal conditions. The results support findings from the previous original English, Spanish, and Turkish versions.

## 1. Introduction

Lower limb problems and dysfunction are an increasing concern in society, regardless of age, culture, and underlying health status [1]. Problems, including pain on movement and at rest plus impaired functions limiting the activities of daily living (ADL) and participation in social life, lead to decreased quality of life [1]. Patient opinions about their own health and functional status may differ from objective evaluations provided by different professionals. Consequently, patient reported outcome measures (PROMs) are recommended to enable the standardization of the data collected and to provide an accurate representation of the patient’s subjective opinions of their functional capabilities [2]. However, the measurements made are only as good as the tools that are used. Further, the clinimetric considerations of both the psychometric and practical properties must both be fully investigated using international guidelines such as Consensus-based Standards for the selection of health status Measurement Instruments (COSMIN) [3].

In English-speaking countries, many PROMs have been created that can be used to assess the functional status of specific joints, conditions, or region-specific conditions [4,5]. However, in Poland, only a limited number of questionnaires are available to assess the function of the lower limbs. Foreign language PROMs must be adapted according to the methodology available in the scientific literature. Particular examples are available for the knees—Knee Injury and Osteoarthritis Outcome Score—KOOS [6,7], Knee Outcome Survey Activities of Daily Living Scale—KOS-ADLS [8], and Lysholm’s scale and International Knee Documentation Committee—IKDC [9]. Recently, a comprehensive Polish validation of the Hip disability and Osteoarthritis Outcome Score (HOOS) was completed [10,11]. Further, the Western Ontario and McMaster Universities Osteoarthritis Index (WOMAC) has been validated for degenerative changes in the hip and knee with WOMAC version-3.1, which is available from the authors’ website (http://www.auscan.org/womac, page last updated: July 2021) in >80 languages, including Polish [12]. However, none of these PROMs are applicable to the whole lower limb as a single kinetic chain that enables the use of a single PROM for all joints and conditions. Only the Lower Extremity Functional Scale (LEFS) [13] and, more recently, the Lower Limb Functional Index (LLFI) [14] are validated for regional use, with neither being available in the Polish language.

The LLFI was developed to assess function within the domains based on the World Health Organization (WHO) International Classification of Functioning, Disability and Health (ICF). The items in the questionnaire contain statements that include body functions or structures and activities or participation in family or social life, which may be affected by lower limb-related problems. This item selection is detailed in-depth within the E-appendices of the publication [14]. The original English version was validated among 127 participants with different lower-limb musculoskeletal conditions that affected particular joints or regions of the lower limb, and involved different conditions and acute, subacute, or chronic phases. The LLFI has demonstrated strong clinimetric properties, including the psychometric characteristics of internal consistency, reliability, error measurement, validity, and responsiveness. The practical characteristics demonstrated brevity, ready transferability to a 100-point scale, ease and rapid scoring and completion by therapists and patients, a low rate of missing responses, and suitable readability, all of which were preferable to the LEFS [14]. These clinimetric properties were reinforced with the scale’s adaptation to Spanish [15] and Turkish [16]. The purpose of this study was to perform a translation and cross-cultural adaptation of the LLFI to establish a Polish version (LLFI-PL) (see Appendix A) and to evaluate its psychometric properties.

## 2. Materials and Methods

### 2.1. Subjects

Patients were recruited between January and May 2018 from a sample of convenience of all patient visits with a specialist rehabilitation physician or orthopedic surgeon at the Specialist Hospital in Rudna Mała, Poland. Inclusion criteria were being diagnosis by a specialist based on an interview, a physical examination, and imaging studies as required (e.g., USG, MRI, CT, X-ray); being more than 18 years age, a native speaker of Polish, having a symptoms duration greater than 4 weeks, having provided informed consent, and diseases/injuries located in the lower limb. This provided a sample of patients with various lower limb conditions, which included osteoarthritis; arthroplasty due to osteoarthritis; injuries to muscles, nerves, ligaments, menisci, bones, and joints; contusion/hematoma; patellar chondromalacia; and joint deformations. Exclusion criteria were coexisting neurological disease, failure to provide written consent, and an inability to read Polish.

### 2.2. Ethics Approval

The Bioethical Commission of the University of Rzeszów granted permission to conduct the present research (resolution no. 2017/06/31b). Informed consent was written and obtained from all participants.

### 2.3. Design

This was a two-stage, cross-sectional study with the repeated measures of two variables during re-test examination.

**Figure 1:**
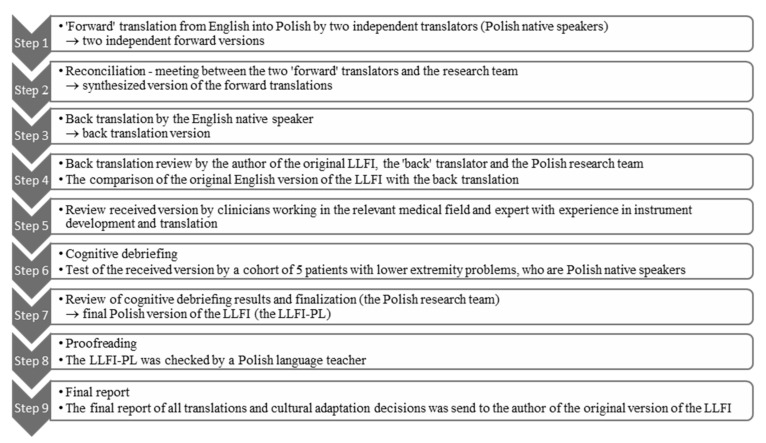
Flow chart of the translation and cultural adaptation process of the Lower Limb Functional Index from English to Polish.

*Stage 1* involved the translation and cross-cultural adaptation of the LLFI into Polish. This was completed in accordance with the International Society for Pharmacoeconomics and Outcomes Research (ISPOR) guidelines and was approved by regulatory agencies such as the Food and Drug Administration and the European Medicines Agency [17]. This consisted of nine steps, each of which was documented with a written report (Figure 1).

Step 1. Two forward translations were performed by two independent translators whose native language is Polish. This process allows Polish language equivalents to be introduced in place of terms that are otherwise difficult to translate.

Step 2. A reconciliation meeting between the two forward translators and the authors of the Polish adaptation to create a common version from the two forward translations.

Step 3. Back translation by an independent English native speaker who is fluent in Polish and who was not familiar with the original LLFI version.

Step 4. Back translation review of the English back translation version was compared to the original English version by the author of the original LLFI, the “back” translator, and the authors of the Polish adaptation.

Step 5. Review by clinicians working in the relevant medical field who were bilingual. The draft LLFI-PL version of the questionnaire was assessed by a three-person expert panel: an orthopedic surgeon, one physician specialized in rehabilitation medicine, and a physiotherapist with expertise in instrument development and translation. The panel qualitatively assessed compliance using a 5–0 Likert-scale (5 = full compliance to 0 = non-compliance) for each question in the source version with the relevant question in the Polish version.

Step 6. Cognitive debriefing with the draft version pilot-tested on a group of five symptomatic patients with lower extremity problems that were present for >3 months. This assessed the face and content validity through the accuracy of questions and the clarity of the wording. The group assessed whether a given position of the scale was fully understood or if it raised doubts using a three-point (2,1,0) scale where: 2 = completely understood, 1 = partially understood, and 0 = completely incomprehensible. In the event that the question was incomprehensible to the respondent, they were asked to indicate the reason for the lack of understanding.

Step 7. Review of cognitive debriefing results and finalization to create a final Polish version, the LLFI-PL.

Step 8. Proof reading by a Polish language teacher to check the LLFI-PL for any errors (spelling, grammatical), which could have occurred during the translation process.

Step 9. A final report that provided a description of all translations and cultural adaptation decisions was sent to the author of the original version of the LLFI.

*Stage 2* involved a prospective evaluation of the essential psychometric properties of the LLFI-PL. The subjects were evaluated twice with an initial baseline examination that consisted of the respondents completing the Polish versions of all of the questionnaires: the LLFI-PL, the WOMAC, the EuroQol 5-Dimensions 5-level version questionnaire (EQ-5D-5L), and an 11-point Pain Numerical Rating Scale (P-NRS), which was anchored at 0 = No Pain to 10 = Worst Pain Possible. During the second examination (re-test), following a period of non-treatment, patients completed the LLFI-PL and the P-NRS. This was at 3–7 days (average = six days) after the first examination, which is considered to be adequate and reasonable [3,18]. The P-NRS completion eliminates unstable participants from reliability analysis for whom the pain difference between the baseline and the retest was >+/−1 point. There is a low probability of changes in symptoms during this period, and the recollection of the original responses is reduced [19].

### 2.4. Research Tools

#### 2.4.1. The Lower Limb Functional Index

The LLFI assesses the impact of any lower limb problem on everyday activities. It is a 25-item regional PROM with a three-point response option of “Yes” (points = 1), “Partly” (points = 1/2), and “No” (points = 0), with a raw score range of 0–25 points. The final score is calculated by the simple addition of the responses from the 25 items. The sum is multiplied by four and then subtracted from 100 to generate a 0–100% score (100% = no disability/normal function) [14].

#### 2.4.2. The Western Ontario and McMaster Universities Osteoarthritis Index

The WOMAC v.3.1 was used to subjectively assess the functional status of patients. It includes 24 questions on a five-point (0–4) scale that determines symptom intensity in three domains: pain (five items), stiffness (two items), and function (17 items). The data can be standardized to a range of values from 0–100 on a percentage scale (0 = worst health status to 100 = best health status). The WOMAC has been adapted into multiple languages, including Polish [12].

#### 2.4.3. The EuroQol Health Questionnaire 5-Dimensions 5-Level

The EQ-5D-5L consists of two parts. The questions from the initial part are grouped into five life-domains: movement, self-service, everyday activities, pain/discomfort, and emotional state. Each question is assessed on five levels, from 1 = minimum (no problems/no pain) to 5 = maximum (impossible to perform/max pain). The second part consists of a 0–100-point visual analogue scale (VAS), where 0 = minimum (worst health status) to 100 = maximum (best health status). A basic subdivision can be made according to the structure of the EQ-5D-5L, presenting results from the EQ-index value (1–5, the higher the score, the worse the condition) and presenting results of the EQ-VAS as a measure of overall self-rated health status (0–100, the higher the score, the better health status). The questionnaire has been adapted to a Polish version [20].

#### 2.4.4. The 11-Point Pain Numerical Rating Scale (P-NRS)

The 11-point P-NRS was used, where 0 = minimum (no pain) and 10 = maximum (the highest imaginable intensity). The recall period was “last week”, and during the re-assessment, it was specified so that it concerned the period between test and re-test [21].

### 2.5. Statistical Analysis

All statistical analyses were conducted using SPSS Statistics software version 24. The level of statistical significance was assumed to be *p* < 0.05. The normal distribution of the results of this study was verified using the Kolmogorov–Smirnov test.

#### 2.5.1. Internal Consistency

The internal consistency was determined using Cronbach’s alpha (α) coefficient, where α should be between 0.70 and <0.95 [18]. Data from the first examination, at baseline, were included in the analysis (*n* = 125).

#### 2.5.2. Test–Retest Reliability

The intra class correlation (ICC_2.1_, CI = 95%) was used to assess test–retest reliability in patients who had completed the LLFI-PL twice and for whom the difference on the P-NRS between the baseline and retest period was +/−1 point (stable status between test and retest, *n* = 94). In addition, the Pearson’s correlation coefficient (PCC) was also determined between the two LLFI-PL measurements. Fisher’s F test was used to assess the statistical significance of the PCC. Reliability was good when the ICC and PCC were *r* ≥ 0.70 [22].

#### 2.5.3. Measurement Error

To assess error the Standard Error of Measurement (SEM) and the Minimal Detectable Change at the 90% level (MDC_90_), the following formula was used: SEM = [SD√(1-R)], where SD = standard deviation of the measurement, and *r* = test–retest reliability coefficient; MDC_90_ then uses the relevant z-value [MDC_90_ = SEM × 1.65 × √2]. The sample included all participants who completed the LLFI-PL at baseline and the reassessment period and for whom the difference on the P-NRS between the baseline and retest periods was +/−1 point (*n* = 94) [19].

#### 2.5.4. Construct Validity

To evaluate the construct validity, the PCC was calculated between the LLFI-PL, the WOMAC (total, pain, stiffness, function), the EQ-5D-5L index value, the EQ-5D-5L-VAS, and the P-NRS (*n* = 125). Consequently, the LLFI-PL should correlate highly with the WOMAC, which is also used to assess the function of the joints of the lower extremities. It should correlate moderately with the EQ-5D-5L index value because the generic questionnaire was designed to measure both the functional state, pain, and the emotional state. The LLFI-PL should correlate moderately with the EQ-5D-5L VAS because this part of the questionnaire assesses the overall sense of health. The LLFI-PL should also correlate moderately with the P-NRS, as it only assesses the pain severity.

Therefore, the a priori hypotheses (7) were proposed as follows:

1a–d. The LLFI-PL should correlate highly with the WOMAC total and with each domain (pain, function, and stiffness).

2–4. The LLFI-PL should correlate moderately with the EQ-5D-5L index value, the EQ-5D-5L VAS, and the P-NRS.

If fewer than 25% of the hypotheses were rejected, the construct validity of the LLFI-PL was considered high. Moderate validity required a rejection rate of 25–50%, and for low validity the rejection rate required >50%. In both latter cases, the LLFI-PL would need to be rejected [18]. The indications for the PCC r strength for validity were < 0.30 = low, 0.30–0.70 = moderate, and ≥0.70 = high [22].

#### 2.5.5. Factor Structure

Exploratory factor analysis (EFA) was used with the maximum likelihood extraction (MLE) and Varimax rotation. The Kaiser–Meyer–Olkin measure of the sampling adequacy (KMO) was set at 0.80–1.0 to indicate adequate sampling, and the significance level of the Barlett Test of Sphericity was *p* < 0.001, indicating that the EFA could be used for data analysis. A single-factor structure was indicated if the extraction requirements satisfied all three a priori criteria:

(1) “Scree plot” inflection at the second point; (2) an eigenvalue >1.0; and (3) variance >10% for a population >100. The item loading was also calculated for the one-factor solution using the MLE method [23].

#### 2.5.6. Practical Characteristics

Readability was performed qualitatively as part of the face and content validity. The completion and scoring times were calculated from the average of the three separate measures of *n* = 15 participants performed by *n* = 15 therapists.

#### 2.5.7. Sample Size

The sample size was pre-selected based on a literature review concerning the creation of the LLFI questionnaire and other language validations [14,15,16]. Post hoc analysis of the test effectiveness was conducted using the ICC with the null hypothesis ICC = 0.7 and a sample group size of 125 people. The estimated ICC value for the Polish population is 0.05. The accuracy of the test is extremely high, showing over 0.999 for the total score. This shows that the sample group size was satisfactory.

## 3. Results

### 3.1. Stage 1 LLFI Translation and Cross-Cultural Adaptation

As a result of the translation and cross-cultural adaptation of the LLFI to the Polish version, changes were introduced at several stages of the process as follows:

Step 2. The reconciliation meeting determined some acceptable differences between the two forward translations, resulting from the many Polish language equivalents that could have been used by the translators.

Step 4. The back translation review agreed that a change be made to item #24. A minor problem was with the phrase “unaccustomed footwear” and matching its best meaning in Polish to the concept item approximated to “shoes that I am not used to”.

Step 5. The review by bilingual clinicians from relevant medical fields determined that from the panel’s results, four questions were distinguished with minor comments (#1, 5, 11, and 15), and subsequent corrections were made.

Step 6. The cognitive debriefing, which analyzed the five symptomatic respondents’ responses (average = 2.0/2.0), indicated patient cognitive acceptance, and no corrections were required.

Step 7. The review of the cognitive debriefing results and finalization saw no changes to the LLFI-PL, and the final version approved.

Step 8. Proof reading indicated no errors and resulted in the consequent adoption of the LLFI-Pl.

This translation and cross-cultural adaptation process produced the LLFI-PL (Appendix A).

### 3.2. Stage 2 Psychometric Investigation

#### 3.2.1. The Clinical Characteristics of the Patients

A total patient sample of *n* = 125 qualified for the study: 69% of the patients who were contacted, age $\overset{-}{x}$ = 52.9±19.6 years, range 20–87, 56% female, symptoms duration $\overset{-}{x}$ = 17.7 ± 18.4 weeks, range 5–71 weeks.

The characteristics of problems, history, diagnosis, and affected area are presented in Table 1.

#### 3.2.2. The Research Tools Absolute Values

The absolute values of the PROMs are presented in Table 2.

#### 3.2.3. Internal Consistency

The internal consistency of the LLFI-PL was within the recognized acceptable range with Cronbach’s α = 0.936 (*n* = 125) (Table 3).

#### 3.2.4. Test–Retest Reliability and Measurement Error

The value of ICC_2.1_ was excellent (0.962, CI ranged from 0.941–0.975, *n* = 94). The SEM = 4.83%, and MDC 90%CI = 11.3%. The SEM calculated for 50% (*n* = 47) of the subject pool with a more consistent baseline severity (73–100% LLFI-PL) was 1.69%, and the MDC 90%CI was 3.93% (Table 3). In addition, the correlations (PCC) between the two LLFI-PL measurements were also high *r* = 0.843 (*p* < 0.0001, Fisher’s F test), which further indicated good test–retest reliability.

#### 3.2.5. Construct Validity

The LLFI-PL construct validity was assessed using the PCC and the reference questionnaires (*n* = 125). The LLFI-PL correlated strongly with the WOMAC and the subscales of pain and function and correlated moderately with the subscales for stiffness. Correlation was moderate with the EQ-5D-5L (index value), the EQ-5D-5L-VAS, and with the P-NRS. Consequently, all proposed a-priori hypotheses were accepted, except for 1d (LLFI-PL correlation with the WOMAC stiffness domain was moderate not high), indicating high construct validity (Table 4).

#### 3.2.6. Factor Structure

The EFA was conducted (*n* = 125) to assess factor structure and to indicate construct validity. Initially, the factor analysis was performed without a single-factor extraction option. The KMO test was adequate (0.88), and Bartlett’s Test of Sphericity was significant (*p* < 0.0001).

**Figure 2:**
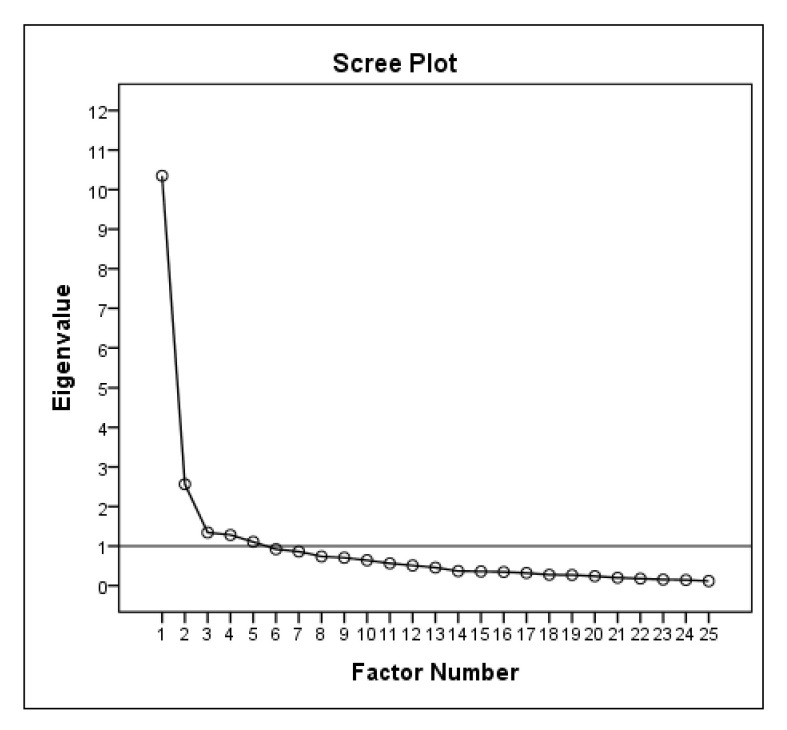
Scree plot with a horizontal line at eigenvalue = 1.0.

A total of five factors were extracted from the raw data analysis with eigenvalues > 1 (Figure 2, horizontal line indicates eigenvalue = 1). However, only the single-factor solution fit all three a priori assumptions, which complied with the a priori requirements for a single-factor structure (Table 5). The item loading for the one-factor solution for the MLE method is shown in Table 6. Items 22, 23, 9, 19, and 20 were the least embedded in the determined single factor.

#### 3.2.7. Practical Considerations

The time to complete the questionnaire was 172 ± 33 s, and scoring was 20 ± 9 s. Missing responses were minimal and only occurred with items 3, 5, 11, and 18, which each missed once in four separate responses. Respondents did not indicate that these items were missed due to issues with understanding the question or item comprehension. Consequently, no further corrections to the LLFI-PL were necessary.

## 4. Discussion

The United States FDA defines PROMs as “any report of the patient’s health condition that comes directly from the patient, without interpretation of the patient’s response by a clinician or anyone else” [24]. A comprehensive assessment of patient health status should, consequently, combine objective data with the patient’s subjective opinion [8].

The cultural and linguistic adaptation that produced the LLFI-PL complied with recognized standards [17]. This ensured the linguistic proportionality of the concepts used and accounted for the slight discrepancies from a number of synonyms for individual words. The majority of proposed hypotheses were proven. The LLFI-PL demonstrated high but suitable and not excessive internal consistency and test–retest reliability. The expected correlations for construct validity were confirmed, as only one was rejected, which satisfied the adopted criteria [18] for high construct validity. The EFA of the LLFI-PL confirmed a single-factor structure, though the inflection at point 2 in the scree plot is at an Eigenvalue >1.0, which accounts for <10% variance. This suggests the potential that a modification to the questionnaire could be made, such as shortening the questionnaire to remove items and to consolidate the factor structure. This would support the internal consistency value findings of 0.936, approaching the upper threshold of α ≤ 0.95, though still within acceptable limits. This is consistent with the recommendations of previous authors [14,15,16].

The decision to complete this study was justified, as it provided a regional lower limb PROM in the Polish language that could be applied to a wide range of patients with various functional problems in the lower limbs of varying severity and duration. These findings support those of earlier linguistic studies previously mentioned and support the role of regional PROMs, such as the LLFI, to be integrated into clinical practice and scientific research projects to better assess the function of the lower limbs.

This study’s results demonstrated the LLFI-PL has comparable psychometric properties to the original English, Spanish, and Turkish versions [14,15,16]. The internal consistency (α = 0.94) is slightly higher than the original and Spanish versions (α = 0.91) [14,15] and notably higher consistency than the Turkish version (α = 0.82) [16]. This is most likely due to the culturally distinct response attitudes of populations in the Turkish region, as similar findings are also found for Persian responses to questionnaires [25,26].

The test–retest reliability (ICC_2.1_=0.96, at 6 days on average) is identical to the Spanish version (ICC_2.1_ = 0.96, at 7 days) [15] and is comparable to both the original and Turkish versions (at three days, ICC_2.1_ = 0.97) [14,16].

The LLFI-PL error scores (SEM = 4.8%, MDC_90_ = 11.3%) are slightly higher than the three published versions: the original English (SEM = 2.8%, MDC_90_ = 6.6%) [14], Turkish (SEM = 3.2%, MDC_90_ = 5.8%) [16], and Spanish (SEM = 3.1%, MDC_90_ = 7.1%) [15] versions. As SEM is dependent on the baseline SD, a diverse baseline level of severity due to symptom severity levels would increase the SD value and the subsequent SEM and MDC. Consequently, reanalysis of a sub-population of 50 % of subjects (*n* = 47) with the highest level of function on the LLFI-PL, produced results that are comparable to other language versions for the SEM–1.69% and for the MDC90–3.93%.

The construct validity, assessed with the PCC, was higher with the joint and condition specific WOMAC total (*r* = 0.81) than with the generic EQ-5D-5L (*r* = −0.63), the EQ-5D-5L-VAS (*r* = 0.57), and the P-NRS (*r* = 0.39). These correlation differences were expected with the higher level due to the greater relevance and specificity of a joint/condition-related PROM compared to a general health and quality of life PROM or an 11-point P-NRS. These are mildly higher than the Spanish findings for the WOMAC (PCC, *r* = 0.77), EQ-5D-3L (*r* = 0.62) and the EQ-5D-3L-VAS (*r* = 0.58) [15] and are similar to the Turkish findings, where the SF-36 subscales were used and which had a high-moderate finding for the physical dimensions (from *r* = 0.43 to *r* = 0.76) but moderate-low (from *r* = 0.20 to *r* = 0.66) for the mental dimension [16].

The factor structure finding in versions of the LLFI (English, Spanish, and Turkish) recommend a preferred single-factor structure. This was achieved consistently with the recommended MLE and Varimax rotation format [14,15,16]. The EFA of the LLFI-PL confirmed a single-factor structure, despite the presence of the inflection at point 2 in the scree plot, whereas point #2 at an E = 2.034 exceeded the arbitrary Eigenvalue 1.0 cutoff. However, at 8.14% this remains as counting for <10% variance, the recognized required minimum to contribute to an additional factor under the EFA a priori determination. This finding suggests that a modification to the questionnaire may be possible to improve the practicality, such as shortening the questionnaire to remove potential redundant items. This is consistent with the recommendations of previous authors [14,15,16]. In recent work, a shortened ten-item version of the LLFI retained the essential psychometric properties of the original measure while improving factor structure and practicality [27]. Some authors suggest that the use of a dual-factor uni-dimensional model may also be present when both Classical Test Theory (CTT–EFA, CFA) and Modern Test Theory (MTT–Rasch Analysis) are considered in tandem [28]. This is particularly so when the CFA suggests a dual-factor structure, while the more simplistic EFA suggests that a single-factor structure is present, as is the case with the LLFI-PL and each of the other versions. The LLFI-PL questionnaire is easy and quick to complete and score. The questions are simple and clearly defined, so the burden on the patient and therapist is minimized. The time needed to complete (172 ± 33 s) and score (20 ± 9 s) the questionnaire is marginally longer than determined by the original study (131 ± 23 and 17 ± 5 s, respectively) [14].

## 5. Limitations and Strengths

The current study limitations include a lack of an assessment for the responsiveness of the LLFI-PL. Further, ideally, a regional criterion such as the LEFS [13] should be used for assessing construct validity, but, as there are none available in Polish, this was not possible. The substitution of the WOMAC was not ideal, but as a PROM that is valid for both the hip and knee, it provided a regional indication.

The study strengths include the use of standardized methods for both the cross-cultural adaptation and assessment of the psychometric properties. Further strengths are the prospective nature, the adequacy of the sample size, and the diversity of the conditions affecting each lower limb sub-region with varied degrees of severity and duration.

## 6. Future Considerations

The lack of determination of the responsiveness suggests that future studies need to consider the ability of the LLFI-PL to detect change in the construct measured over time. Furthermore, it indicates that a larger study population (~250–500) or that the use of data pooling should be used to definitively clarify the factor structure through confirmatory factor analysis (CFA) [29]. Furthermore, a shortened version should be considered and with fully determined clinimetric properties. Other areas of research would include the validation of this PROM in conditions of abnormal muscle coactivation that affect gait performance, such as those found in neurological patients including hereditary spastic paraparesis [30].

## 7. Conclusions

The LLFI-PL is a reliable and valid questionnaire for Polish-speaking patients with lower limb musculoskeletal disorders. The psychometric properties are comparable with the original English version and the published Spanish and Turkish linguistic and cultural adaptations. The LLFI-PL can be used in clinical practice and scientific research projects on patients with various lower limb functional problems and variable levels of severity and symptom duration. Further research is required to clarify the factor structure, its role in neurological patients, the relevance of a shortened ten-item version, and the level of responsiveness.

## Figures and Tables

**Table 1:** The clinical characteristics of the patients.

Subregion	^a^ Diagnosis	*n* (%)
**HIP**	Osteoarthritis Total hip arthroplasty	**42 (33.6)** 38 (90.5) 4 (9.5)
**UPPER LEG**	Muscle strain: Biceps femoris Quadriceps femoris Adductors	**7 (5.6)** 7 (100) 2 (28.6) 3 (42.9) 2 (28.6)
**KNEE**	Osteoarthritis Ligament injury: ACL MCL Patellar chondromalacia Meniscus repair Knee arthroplasty	**53 (42.4)** 27 (50.9) 5 (9.4) 5 (9.4) 2 (3.8) 4 (7.5) 10 (18.9)
**LOWER LEG**	Muscle strain: Triceps surae Tibialis anterior Achilles tendon Injury (contusion/hematoma)	**11 (8.8)** 7 (63.6) 5 (45.5) 1 (9.1) 1 (9.1) 4 (34.4)
**ANKLE**	Injury of ligaments Osteoarthritis	**12 (9.6)** 11 (91.7) 1 (8.3)
**FOOT**	A joint Hallux	**4 (3.2)** 2 (50.0) 2 (50.0)
**WHOLE LIMB**	Neurological reasons	**5 (4.0)** 5 (100.0)
**MULTIPLE AREAS**	Diagnoses were included above	**9 (7.2)**
**PAIN**	Acute ˃ 4–6 weeks	40 (32.0)
**CHARACTERISTIC**	Subacute 6–12 weeks	13 (10.4)
	Chronic ≥ 12 weeks	72 (57.6)

*n =* number, *% =* percent. ^a^ Subregion and percentage values of diagnoses include individuals with multiple (two or more) affected subregions. Consequently, totals are greater than 100%.

**Table 2:** The absolute values of all PROMs.

Questionnaire	$\overset{-}{x}$ ± SD	Me	Range
**LLFI -PL I (0–100)**	60.7 ± 25.1	66.0	6.0–100.0
**LLFI-PL II (0–100)**	66.4 ± 24.4	72	6.0–100.0
**NRS I (0–10)**	5.2 ± 1.8	5	2–9
**NRS II (0–10)**	4.6 ± 1.75	4	1–9
**WOMAC I (0–100)**	61.7 ± 21.6	61.0	16.0–95.0
**EQ-5D-5L Index value I (1–** **5)**	1.7 ± 1.17	1.8	1.1–2.0
**EQ-5D-5L—VAS I (0-100)**	60.2 ± 19.8	60.0	15.0–95.0

*$\overset{-}{x}$* (mean), *SD* (standard deviation), *Me (* median), *LLFI-PL* (Lower Limb Functional Index—Polish version), *I* (test examination), *II* (retest examination), *WOMAC* (Western Ontario and McMaster Osteoarthritis Index), *EQ-5D-5L* (Euro–Quality of Life Questionnaire), *NRS* (Numerical Rating Scale for pain).

**Table 3:** Psychometric properties of the LLFI-PL.

**Questionnaire**	**Internal Consistency *n* = 125**	**Test–Retest Reliability** ***n* = 94**			**Error Score** **(0–100%)** ***n* = 94**		**Error Score** **(73–100%)** ***n* = 47**	
	**Cronbach’s Alpha**	**(ICC_2.1_)**	**95% CI** **LB UB**		**SEM**	**90%CI MDC**	**SEM**	**90%CI MDC**
LLFI-PL	0.936	0.962	0.941	0.975	4.83	11.3	1.69	3.93

*LLFI-PL* Lower Limb Functional Index—Polish version; *ICC* intraclass correlation coefficient; *LB* lower bound; *UB* upper bound; *SEM* standard error of the measurement; *CI* confidence interval; *MDC* minimal detectable change.

**Table 4:** PCC between the LLFI-PL and the WOMAC, the EQ-5D-5L, and the Pain NRS.

Questionnaire	LLFI-PL (*n* = 125)
	PCC
WOMAC Total	*r* = 0.81, *p* < 0.001 *
WOMAC Pain	*r* = 0.77, *p* < 0.001 *
WOMAC Stiffness	*r* = 0.45, *p* < 0.001 *
WOMAC Function	*r* = 0.81, *p* < 0.001 *
EQ-5D-5L Index value	*r* = −0.63, *p* < 0.001 *
EQ-5D-5L—VAS	*r* = 0.57, *p* < 0.001 *
NRS Pain	*r* = −0.39, *p* < 0.001 *

*LLFI-PL* (Lower Limb Functional Index—Polish version), *I* (test examination), *II* (retest examination), *WOMAC* (Western Ontario and McMaster Osteoarthritis Index), *EQ-5D-5L* (Euro–Quality of Life Questionnaire), *NRS* (Numerical Rating Scale for pain), *PCC* (Pearson’s correlation coefficient).

**Table 5:** Sums of load squares after isolation (analysis without one factor option enforced).

Factor	Total	% of Variance	Cumulative %
1	9.919	39.676	39.676
2	2.034	8.136	47.812
3	0.930	3.722	51.534
4	0.960	3.842	55.375
5	0.784	3.136	58.512

**Table 6:** Factor loadings of all items for one factor solution.

LLFI-PL Items	Factor 1
LLFI-PL_7	0825
LLFI-PL_25	0799
LLFI-PL_4	0795
LLFI-PL_16	0785
LLFI-PL_1	0782
LLFI-PL_11	0769
LLFI-PL_18	0759
LLFI-PL_6	0729
LLFI-PL_10	0727
LLFI-PL_13	0724
LLFI-PL_15	0721
LLFI-PL _5	0680
LLFI-PL_24	0661
LLFI-PL_3	0631
LLFI-PL_17	0579
LLFI-PL_14	0501
LLFI-PL_2	0496
LLFI-PL_21	0478
LLFI-PL_12	0475
LLFI-PL_8	0424
LLFI-PL_19	0374
LLFI-PL_20	0371
LLFI-PL_9	0366
LLFI-PL_23	0327
LLFI-PL_22	0,298

*LLFI-PL* (Lower Limb Functional Index—Polish version).

## Data Availability

The data that support the findings of this study are available from the corresponding author upon reasonable request.

## Appendix A

**LOWER LIMB FUNCTIONAL INDEX—WERSJA POLSKA (LLFI—PL)**
**IMIĘ I NAZWISKO: _______________________________ DATA: _______________________________** **URAZ/PROBLEM:__________________________________ LEWA NOGA □ PRAWA NOGA □**

**PROSZĘ UZUPEŁNIĆ**: Problemy z Twoimi nogami mogą utrudniać wykonywanie niektórych czynności. Poniższa lista opisuje typowe problemy z tym związane. Proszę odnieść się do sytuacji z ostatnich kilku dni. **Jeśli któreś stwierdzenie dotyczy Pana/Pani, proszę zaznaczyć kwadracik *„CZĘŚCIOWO”* lub *“TAK”.* Jeśli stwierdzenie Pana/Pani nie dotyczy, proszę zaznaczyć *“NIE”.***

**Z POWODU MOJEJ NOGI / MOICH NÓG:**

**NIE Częściowo TAK**

**0 ½ 1**

☐ ☐ ☐ 1. Pozostaję w domu przez większość czasu.
☐ ☐ ☐ 2. Często zmieniam pozycję dla większego komfortu.
☐ ☐ ☐ 3. Unikam ciężkich prac (np. sprzątania, podnoszenia rzeczy cięższych niż 5 kg, wykonywania prac w ogrodzie itp.).
☐ ☐ ☐ 4. Częściej odpoczywam.
☐ ☐ ☐ 5. Proszę inne osoby, by zrobiły za mnie niektóre czynności.
☐ ☐ ☐ 6. Odczuwam ból /mam problem prawie przez cały czas.
☐ ☐ ☐ 7. Mam trudności z podnoszeniem i noszeniem (np. toreb, zakupów o wadze do 5 kg).
☐ ☐ ☐ 8. Zmienił mi się apetyt.

☐ ☐ ☐ 9. Chodzenie lub rekreacja lub aktywność sportowa jest utrudniona.
☐ ☐ ☐ 10. Mam trudności z normalnymi domowymi lub rodzinnymi obowiązkami i pracami.
☐ ☐ ☐ 11. Gorzej sypiam.
☐ ☐ ☐ 12. Potrzebuję pomocy w samoobsłudze (np. myciu i utrzymaniu higieny).
☐ ☐ ☐ 13. Moja codzienna aktywność jest utrudniona (praca, kontakty społeczne).
☐ ☐ ☐ 14. Łatwiej się irytuję i/lub wpadam w złość.
☐ ☐ ☐ 15. Czuję się słabszy/-a i/lub zesztywniały/-a.
☐ ☐ ☐ 16. Moje niezależne przemieszczanie się jest utrudnione (prowadzenie samochodu, korzystanie z transportu publicznego).

☐ ☐ ☐ 17. Mam trudności lub potrzebuję pomocy w ubieraniu się (np. spodni, bielizny, butów, skarpetek).
☐ ☐ ☐ 18. Mam trudności ze zmienianiem kierunków, skręcaniem lub obracaniem się.
☐ ☐ ☐ 19. Nie jestem w stanie poruszać się tak szybko jak chciałbym/chciałabym.
☐ ☐ ☐ 20. Mam trudności z dłuższym lub przeciągającym się staniem.
☐ ☐ ☐ 21. Mam trudności ze zginaniem się, kucaniem i/lub schylaniem się.
☐ ☐ ☐ 22. Mam trudności z długimi lub przeciągającymi się spacerami.
☐ ☐ ☐ 23. Mam trudności z chodzeniem po schodach.
☐ ☐ ☐ 24. Mam trudności z dłuższym lub przeciągającym się siedzeniem.
☐ ☐ ☐ 25. Mam trudności z utrzymaniem równowagi na nierównej powierzchni i/lub w obuwiu do którego jestem nieprzyzwyczajony/a.

**Wynik LLFI-PL: W celu obliczenia wyniku—dodaj zaznaczone odpowiedzi:** __________WYNIK CAŁKOWITY (punkty LLFI-PL); 100 Skala: 100-(WYNIK CAŁKOWITY × 4) = _________% MDC*(90% CI *): 3,93 % LLFI-PL. Zmiana mniejsza niż podana może wynikać z błędu.

* MDC—Minimalnie Zauważalna Zmiana; * CI—Przedział Ufności; email autora LLFI—Charles Philip Gabel i wsp.: cp.gabel@bigpond.com; email autora adaptacji do wersji polskiej LLFI-PL—Agnieszka Bejer i wsp: agnbej@wp.pl.
